# Private sector initiatives to tackle the burden of COVID-19: experiences from the Nigerian frontline

**DOI:** 10.11604/pamj.2021.38.233.24634

**Published:** 2021-03-04

**Authors:** Akaninyene Otu, Emmanuel Effa, Victor Umoh, Nicholas Maxwell, Andrew Ekpenyong

**Affiliations:** 1 Department of Internal Medicine, College of Medical Sciences, University of Calabar, Calabar, Cross River State, Nigeria,; 2 Joseph Ukpo Hospitals and Research Institutes (JUHRI), Afua Site, Ibiono Ibom, Nigeria,; 3 Foundation for Healthcare Innovation and Development (FHIND), Cross River State, Nigeria,; 4 Department of Infection and Travel Medicine, Leeds Teaching Hospitals NHS Trust, Leeds, United Kingdom,; 5 Department of Internal Medicine, Faculty of Medicine, University of Uyo, Uyo, Akwa Ibom State, Nigeria,; 6 University of Rochester School of Medicine and Dentistry, Rochester, New York, USA,; 7 Department of Physics, Creighton University, NE, 68178, Nebraska, United States of America

**Keywords:** COVID-19, private sector, Nigeria

## Abstract

Across Africa, there is some evidence of COVID-19 private sector activities to tackle COVID-19 which include the development of rapid diagnostic kits, deployment of e-health platforms for bespoke health workforce training, disease surveillance, reporting, auto-screening and advisories. Inequities in living and access to care by disadvantaged populations in the rural areas have been ameliorated by multi-pronged responses such as that mounted by the Joseph Ukpo Hospitals and Research Institute (JUHRI) in Nigeria. The provision, production and donation of personal protective equipment (PPE), the production of hand sanitizers and the engagement of the local community in the process represents an effective strategy to contain COVID-19, protect health workers and provide pathways for economic support for people whose sources of income have been upended during the pandemic. The JUHRI experience underpinned by Catholic medical ethics provides concrete evidence of the value of private sector participation in dealing with public health emergencies.

## Perspectives

**COVID-19: humankind is facing its greatest existential threat:** against the background of the fragile health systems and inadequate health financing in Africa, the need for private sector participation in controlling a public health emergency has never been so dire as with the SARS-CoV-2 (COVID-19) pandemic. COVID-19 has brought the capacity of governments, even of the resource-rich nations, to deal with a global health crisis that has killed well over half a million people [1] into sharp focus. Western economies have grappled with the provision of personal protective equipment (PPE), testing kits, isolation facilities and ventilators while overseeing lockdowns and keeping essentials like food and supplies flowing through organized arrangements [2]. Initially, Africa appeared to be spared the effects of this deadly virus, but the number of COVID-19 cases and deaths has continued to rise steadily following more widespread testing [3]. Africa´s ability to mount a robust response to COVID-19 is hampered by perennial issues such as staff shortages, weak medical supply chains, a dearth of water and sanitation facilities and lack of surge capacity - problems that reflect decades of inadequate health financing and weak health governance [4]. In 2020, COVID-19 left few unaffected with dire impacts on millions across the globe. The economic impact from COVID-19 has disproportionately affected the most vulnerable leading to rising poverty levels, with up to 12,000 people per day facing starvation in 2020 from this pandemic [5]. COVID-19 infections are still rising in 41 countries across the globe with well over 2.2 million reported deaths attributed to this viral infection as of 4^th^ February 2021 [6]. The emergence of more contagious strains of the 2019 novel coronavirus poses new challenges for humankind. In 2020, COVID-19 is estimated to have infected 2.7 million Africans and killed over 65,000 people [7]. With the discovery and rollout of vaccines that confer high levels of protection against COVID-19 [although it is unclear for how long], there appears to be a glimmer of hope. More than 12 billion COVID-19 vaccine doses have been announced for release in 2021 [5] but the logistical challenges of getting these vaccines (which require strict cold chains) to the African continent appear to be very daunting.

**Does the private sector have a role in controlling COVID-19:** there is emerging evidence that Africa is lagging far behind the rest of the world in domestic resource mobilization from private sector engagement [8]. Whilst some African countries are becoming richer, this growth is not being matched by greater spending resulting in persistent (and widening) gaps in health funding [8]. Nigeria´s expenditure in health as a total of government expenditure is still less than 15% and just about 3.7% of GDP. Her public health response to COVID-19 has attracted a lot of global attention. This response has been led by the Federal Government with the Nigeria Centre for Disease Control (NCDC) being at the forefront of COVID-19 testing and surveillance activities. As of 5^th^ February 2021, a total of 1,302,410 COVID-19 diagnostic tests had been carried out on Nigeria´s teeming population of over 200 million people [9]. There were also reports of many healthcare professionals in public and private hospitals across Nigeria absconding from work due to a lack of the appropriate PPE to engage with suspected COVID-19 cases [10,11]. These are reflections of critical gaps in the country´s ability to respond to the numerous challenges posed by COVID-19. The private sector in Nigeria, one of the most affluent in Africa, has risen to support the Nigerian response to COVID-19 [12]. The private sector in Nigeria is estimated to account for up to 90% of Nigeria´s gross domestic product (GDP). On 26^th^ March 2020, the Coalition Against COVID-19 (CACOVID) was formed to mobilize private sector resources to support measures against COVID-19 in Nigeria. There has been a pledge of US $30 million to support the activities of the NCDC from the Nigerian National Petroleum Corporation alongside some oil companies [12]. Many other institutions and individuals have made significant contributions to fighting COVID-19 in Nigeria [13]. With over US $71 million arising from such contributions as of May 2020 [14], Nigeria´s control efforts received a further boost when the Nigeria Catholic Bishops conference offered its 425 health facilities for use as COVID-19 isolation centres [15].

**The Joseph Ukpo Hospitals and Research Institute (JUHRI):** in a bid to promote structured engagement of the private sector with healthcare in Nigeria, the Joseph Ukpo Hospitals and Research Institute (JUHRI) was established in 2018. Consistent with Catholic medical ethics, JUHRI has been working with the government, organizations and individuals who share her vision: that healthcare is a human right and it is our collective responsibility to ensure universal access to it. With the overarching objective of bringing the benefits of modern medical science to those most in need of healthcare, JUHRI has successfully provided 100% free healthcare to a total of 6,570 patients at JUHRI in Afua, Nigeria as of 31^st^ May, 2020. JUHRI´s mobile outreach services that have provided both diagnostic and therapeutic services to thousands of underserved persons have been reported previously [16-18].

**Figure 1:**
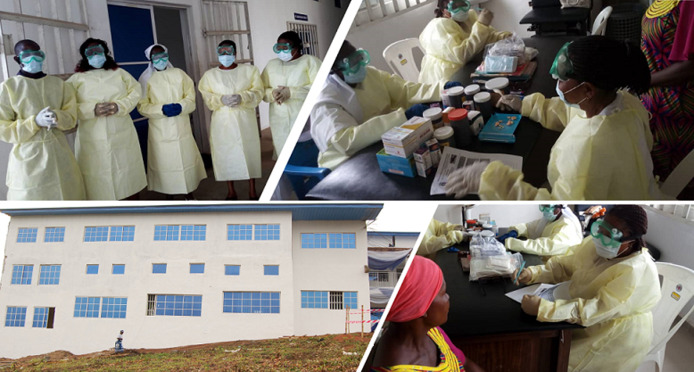
extra PPEs provided to all staff in March 2020; the extra PPEs included water repellent gowns, goggles, face masks, respiratory masks and gloves (top and bottom right pictures); a side relief of JUHRI, Afua site is shown (bottom left)

**Figure 2:**
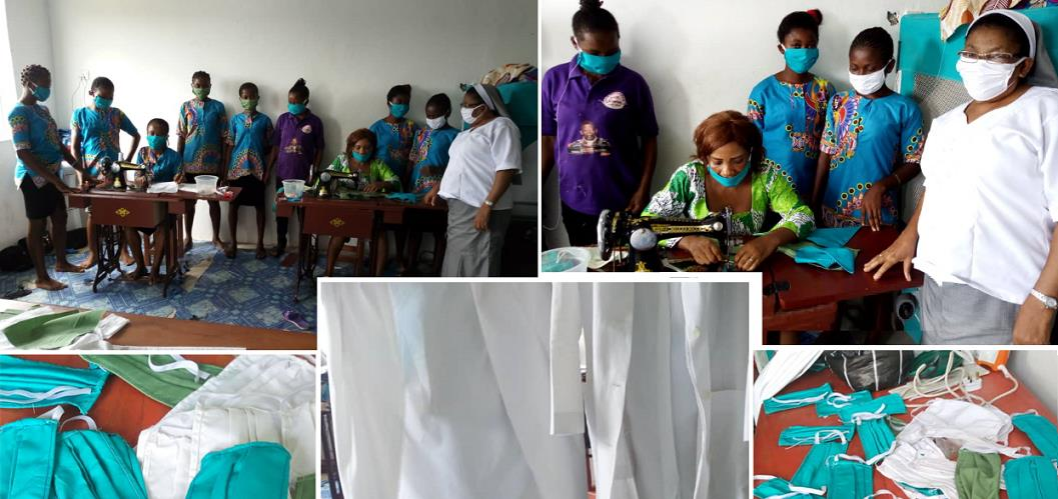
production of PPEs inside JUHRI’s skills acquisition centre

**JUHRI´s multipronged response to COVID-19:** Nigeria´s first case of COVID-19 was an Italian citizen who was diagnosed on February 27, 2020 [19]. Before the index case was diagnosed, the JUHRI management had recognized the potential threat posed by this new virus and taken critical steps to mitigate this by: 1) Providing PPEs to all staff consisting of water repellent gowns, goggles, face masks, and gloves (Figure 1). These PPE were donated to JUHRI by Franciscan Mission Warehouse, Missouri, USA, in 2017. 2) Producing PPEs (cloth masks and clinical coats) locally at JUHRI´s skills acquisition center (Figure 2). Part of JUHRI´s holistic and preventative healthcare strategy is to provide free training in sewing, computer literacy, catering and auxiliary nursing to vulnerable teens and young adults to empower them economically. Nine pioneer students of the center had their sowing training repurposed to produce PPEs including face masks and lab coats. They now have been making about 400 face masks every month, since April 2020. 3) Providing face-masks to all patients regardless of symptoms, since March 31^st^ 2020. 4) Provision of hand sanitizers and extra hand-washing stations on the verandah of JUHRI for immediate use by staff and patients on arrival at the facility. 5) Ensuring appropriate social and physical distancing measures of at least 1.5m amongst patients while waiting for medical attention. 6) Providing daily health education to patients on the dangers of COVID-19 and how to reduce transmission. 7) Donating PPE to nearby hospitals to enhance their COVID-19 preparedness. On a larger scale, JUHRI has acquired SARS-CoV-2 antibody testing kits and supported the government´s COVID-19 control measures by providing free COVID-19 antibody testing in a bid to determine the extent of COVID-19 spread in the Akwa Ibom and Cross River States of Nigeria. Research assistants were trained to facilitate this multi-centre research project. Ethical approvals were obtained from the Health Ethics Committees of University of Calabar and University of Uyo Teaching Hospitals. The antibody testing has been completed and preliminary report shared.

**Applicability to similar contexts/lessons learned:** in the early days of the COVID-19 response, Nigeria like most LMICs lacked a multisectoral containment strategy. This was subsequently instituted by the Presidential Task Force on COVID-19 in collaboration with the NCDC. Most health systems in LMICs are fragile and have poor institutional capacity to initiate and effectively implement the complex multisectoral physical, economic, and behavioural interventions required to contain the COVID-19 outbreak. Often, the national response is driven by a focus on the core elements of an outbreak response including diagnostics, bed capacity, commodity, health workforce and data management [20]. In Nigeria, the public sector has been wholly involved in rolling out and implementing public health interventions. However, stringent limitations were placed on private sector participation in testing and treatment of people who were positive for the disease. Indeed, although there are now guidelines for private sector engagement for testing, this only became available in May 2020 [21]. Useful lessons have been learnt across several countries in Africa. In Ghana, two private sector driven initiatives have added to the efforts of the government in containing the pandemic. These include successful deployment of e-health solutions and teleconsultations as well as the development of rapid diagnostic test kits [22,23]. Again, in Senegal, Bukina Faso and Benin Republic, innovative entrepreneurs have produced PPE (respirators and visors), supported disease surveillance and reporting and setup auto-screening and online advisory applications [24]. These projects, in response to governments´ policies for mitigating the pandemic, buttress the many possibilities that public-private sector synergy can bring. Similar e-health platforms for disease surveillances and health workforce training have also been deployed in approximately 10 States in Nigeria, reaching over 20,000 workers and adoption is increasing rapidly [25]. Arguably, this reduced the bureaucratic red tape prior to roll out and significantly cut the cost associated with organizing face to face training. The Nigerian national government´s leveraging of private sector funding to support the response has largely cushioned the negative impact that the absence of such unplanned extra expenditure would have had on the health system. Given the inequities in access to care by disadvantaged populations in the rural areas, JUHRI´s multi-pronged response represents an effective strategy to contain COVID-19, protect health workers and provide pathways for economic support for people whose sources of income have been upended during the pandemic. Significantly, private sector involvement in the local production and donation of PPEs and hand sanitizers may have lessened the burden on the government of providing these commodities to all health facilities.

## Conclusion

Structured engagement of the private sector could potentially mobilize the necessary capital and harness innovations to mitigate the COVID-19 pandemic in Nigeria and similar settings. The magnitude of the task of providing workable solutions for COVID-19 such as vaccination requires bold collective action from the private sector, multinational organizations and governments. The JUHRI experience provides concrete evidence of the value of private sector participation in dealing with public health emergencies.

**General recommendations on how the private sector can contribute to curbing the COVID-19 pandemic:** i) Partner with governments and communities to provide a multisectoral response to the COVID-19 pandemic. ii) Explore ways of producing and distributing sanitizers, face masks and vital medical supplies needed to tackle COVID-19. iii) Contribute essential products including food, manpower, equipment, transportation and logistics to support the pandemic control. iv) Provide economic initiatives to cushion the financial shocks arising from COVID-19. v) Establish services to protect people’s mental health and provide moral support for all through this pandemic.

## References

[1] Johns Hopkins University Corona virus resource center, COVID-19 dashboard by the Center for Systems Science and Engineering (CSSE) at Johns Hopkins University.

[2] UNDP 6 lessons from China's Zhejiang Province and Hangzhou on how countries can prevent and rebound from an epidemic like COVID-19.

[3] Otu A, Ebenso B, Labonte R, Yaya S (2020). Tackling COVID-19: can the African continent play the long game?. J Glob Health.

[4] Olu O, Drameh-Avognon P, Asamoah-Odei E, Kasolo F, Valdez T, Kabaniha G (2019). Community participation and private sector engagement are fundamental to achieving universal health coverage and health security in Africa: reflections from the second Africa health forum. BMC Proc.

[5] World Economic Forum Is the world up to the challenge of mass COVID-19 vaccination?.

[6] Reuters COVID-19 global tracker.

[7] Africa Center for Strategic Studies Analysing Africa´s second wave of COVID-19.

[8] Reliefweb A heavy burden: the indirect cost of illness in Africa.

[9] National Centre for Disease Control website. COVID-19 Nigeria.

[10] All Africa; daily trust Nigeria: lack of protective equipment - doctors 'absconding' from hospitals over COVID-19.

[11] DW COVID-19: Africa's health workers at risk.

[12] Africa Business Nigerian private sector donates more than most other African countries in fight against COVID-19.

[13] CNBC Africa Nigeria´s private sector coalition raises N15.3bn to fight COVID-19.

[14] The Guardian FG declares N1.689b as public support donations for COVID-19.

[15] The Cable Catholic Church donates all its 425 hospitals in Nigeria as COVID-19 isolation centres.

[16] Ikpeme A, Ani N, Ago B, Effa E, Kosoko-Lasaki O, Ekpenyong A (2017). The value of mobile ultrasound services in rural communities in South-South Nigeria. Open Access Maced J Med Sci.

[17] Ikpeme A, Ani N, Isewele E, Ekpenyong AE, Ekanem E (2019). Risk factors for cardiovascular events in rural Nigeria: a cross sectional survey and review of the current literature. Innovative Journal of Medical and Health Sciences.

[18] Ikpeme A, Ani N, Odusolu P, Eyong E, Ekpenyong AE (2017). Spectrum of disease and diagnostic value of ultrasound in inmates of a correctional facility in Nigeria. European Journal of Pharmaceutical and Medical Research.

[19] Ebenso B, Otu A (2020). Can Nigeria contain the COVID-19 outbreak using lessons from recent epidemics?. Lancet Glob Health.

[20] Nimako BA, Baiden F, Awoonor-William JK (2020). Towards effective participation of the private health sector in Ghana's COVID-19 response. Pan Afr Med J.

[21] Citi newsroom KNUST, Incas develop rapid diagnostic test kit for detecting COVID-19.

[22] Hyaho Medical Centre Nyaho medical centre partners clearspace to launch COVID-19 assessment tool.

[23] Nigeria Centre for Disease Control Integration of private sector laboratories in national COVID-19 response May 2020.

[24] Policy Center for the New South International development and the private sector in the coronavirus outbreak.

[25] InStrat´s Android COVID-19 application.

